# Microbiological Profile of Infectious Keratitis in a Portuguese Tertiary Centre

**DOI:** 10.1155/2019/6328058

**Published:** 2019-10-24

**Authors:** Cláudia Oliveira-Ferreira, Mariana Leuzinger-Dias, João Tavares-Ferreira, Luís Torrão, F. Falcão-Reis

**Affiliations:** ^1^Ophthalmology Department, Centro Hospitalar São João, Porto, Portugal; ^2^Department of Surgery and Physiology, Faculty of Medicine of Porto University, Porto, Portugal

## Abstract

The microbiological profile of infectious keratitis has shown great differences across the world. Due to the continuous shifting trends in microbiological profile and antibiotic resistance patterns reported in several studies, constant local updates are crucial to provide an adequate treatment. The propose of this study was to analyze the incidence of infectious keratitis, possible changing trends in microbiological profile, and bacteria sensitivity to commonly used antibiotics, in our tertiary center, in the last 10 years. A retrospective study was performed, based on the survey review of electronic medical records of all patients with presumed infectious keratitis, between January 1, 2009, and December 31, 2018. Microbial cultures were performed, and patients were treated according to an internal protocol. A total of 1360 samples were included. We obtained a 35.1% culture-positive rate. Bacteria accounted for 76.78% of all positive scrapes (53.34% were Gram positive and 23.44% were Gram negative), *Acanthamoeba* for 12.13%, fungi for 8.16%, and virus for 2.93%. The most frequent agent identified was *Corynebacterium macginleyi* (18.41%), followed by *Staphylococcus aureus* (17.78%), *Streptococcus pneumoniae* (9.41%), and *Pseudomonas aeruginosa* (9.00%). We identified at least one ophthalmologic risk factor in 410 patients (85.77%). Trauma and contact lens wear were the most common risk factors found, accounting for 34.94% (*n* = 167) and 33.47% (*n* = 160) of cases. Sensitivity to fluoroquinolones and aminoglycosides was tested in all bacterial isolates, presenting values of 96.66% and 98.12%. In our region, the most common bacteria are *Staphylococcus aureus*, *Streptococcus pneumoniae*, *and Pseudomonas aeruginosa*, and they showed high sensitivity rates to first-line antibiotics, without any modification or emergence of antibiotic resistance trends during the 10 years of the study. For this reason, we decided to maintain the same internal protocol in our tertiary centre.

## 1. Introduction

Infectious keratitis is a serious and frequent cause of ophthalmology consultation and one of the greatest causes of visual impairment worldwide [1, 2]. The incidence of this pathology is highly variable around the world. It is estimated to affect about 11/100,000 inhabitants in the United States, rising to 799/100,000 inhabitants in developing countries like Nepal [3, 4].

Infectious keratitis is associated with significant morbidity, carrying a high risk of corneal scar, corneal perforation, endophthalmitis, and vision loss.

Contact lens usage and ocular trauma are the major risk factors to develop infectious keratitis. However, other conditions such as ocular surface diseases (blepharitis and dry eye) and systemic disorders (diabetes, rheumatoid arthritis, and acquired immunodeficiency syndrome) are also strongly associated with the onset of this disease [5].

Clinically, it manifests as eye pain, photophobia, blurred vision, and ciliary injection.

The microbiological profile of infectious keratitis has shown great differences across the world, but bacteria triumph as the leading cause of infectious keratitis, followed by fungi (especially in cases of ocular trauma with vegetable matter). Virus and protozoa are less frequent agents [6, 7].

Having in mind this etiological pattern, broad-spectrum antibiotics are used as empiric first-line treatment for presumed infectious keratitis, after obtaining corneal scrapes [2]. However, to maintain the effectiveness of empiric therapy, it is necessary to assure low resistance rates to the selected antibiotics. Due to the widespread use of broad-spectrum antibiotics, and continuous shifting trends in microbiological profile and antibiotics resistance profiles reported in several studies, constant local updates of microbiological profile and antibiotic resistance patterns are crucial to provide an adequate treatment [8–13].

The propose of this study was to analyze the incidence of infectious keratitis, possible changing trends in microbiological profile, and bacteria sensitivity to commonly used antibiotics, in our tertiary center, in the last 10 years.

## 2. Materials and Methods

This retrospective study was performed in a Portuguese tertiary center, based on the survey review of electronic medical records of all patients with presumed infectious keratitis, between January 1, 2009, and December 31, 2018. Presumed infectious keratitis was defined by the presence of a corneal infiltrate >1 mm^2^ in size with or without epithelial defect.

All samples were collected in the emergency room, under topical anesthesia. Every time, scrapes were obtained from two different locations (conjunctival fornix and ulcer base) and then inoculated onto Amies Agar Gel (Copan, Brescia, Italy). Finally, they were sent to the Microbiology Department for culture and antibiotic sensitivity testing. In clinically suspicious or nonresponsive cases, selective media and stains for aerobic and anaerobic bacteria, *Mycobacterium* and *Acanthamoeba*, were used.

Patients were treated according to an internal protocol, using an empiric third- or fourth-generation fluoroquinolone and an aminoglycoside, hourly. If a fungi etiology was suspected (soil contamination or trauma with vegetable matter), topical clotrimazole was added to the treatment regimen.

Patients were assessed for age, gender, presence of risk factors (contact lens usage, ocular trauma, ocular surface diseases, or systemic diseases), sample results, and antibiotic sensitivity test results. The study was therefore divided into 2 periods for analysis: from 2009 to 2013 and from 2013 to 2018.

Statistical analysis was performed using SPSS software, version 22 (IBM, Chicago, IL). To compare differences between groups, chi-square test, *t*-test, logistic, and multiple regression were performed, as appropriated. *P* < 0.05 was considered significant.

## 3. Results

A total of one thousand three hundred and sixty (*n* = 1360) samples were included. Patients' mean age was 46.88 ± 20.327 years (range 2–98), and 50.3% of them were males. Over the years, we observed an increase in the number of suspected cases of infectious keratitis, with the value doubling in 10 years. We obtained a 35.1% (*n* = 478/1360) culture-positive rate. The percentage of positive microbiological cultures per year ranged from 31.31 to 42.22%. Per period, this value was 37.76% and 33.71% in 2009–2013 and 2014–2018, respectively, without significant statistical differences (*P* > 0.05).

**Figure 1:**
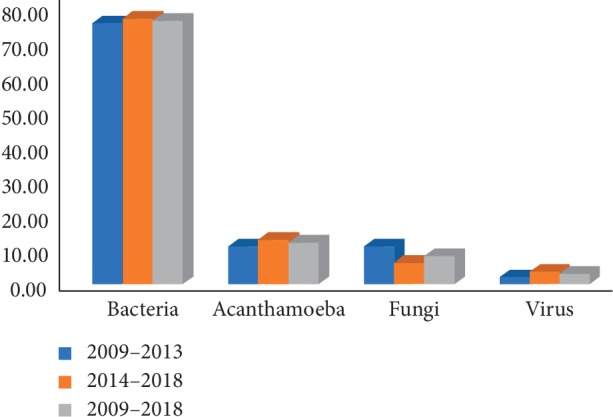
Distribution of microbial profile in the two periods studied.

Bacteria accounted for 76.78% (76% in 2009–2013; 77.40% in 2014–2018) of all positive scrapes (53.34% were Gram positive and 23.44% were Gram negative), *Acanthamoeba* for 12.13%, fungi for 8.16%, and virus for 2.93% (Figure 1).

**Figure 2:**
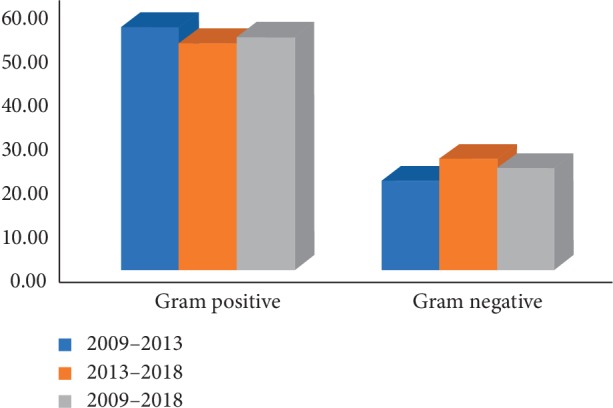
Proportion of Gram-positive and Gram-negative isolates in the two periods studied.

Gram-positive bacteria accounted for 55.50% of all positive scrapes in 2009–2013 and 51.80% in 2014–2018, while Gram-negative bacteria accounted for 20.50% and 25.54% in 2009–2013 and 2014–2018, respectively, without significant statistical changes (Figure 2). The most frequent Gram-positive microorganism isolated was *Corynebacterium macginleyi* (18.41%), followed by *Staphylococcus aureus* (17.78%), *Streptococcus pneumonia* (9.41%), and *Staphylococcus epidermidis* (4.39%). No significant statistical changes were observed in the microbiological profile in both periods. The most frequent Gram-negative agents were *Pseudomonas aeruginosa*, *Serratia* spp., *Moraxella* spp., and *Haemophilus influenzae* (9.00%, 5.65%, 4.18%, and 1.46%, respectively). Again, no significant statistical changes were observed in the microbiological profile in both periods.

*Acanthamoeba* accounted for 11% and 12.95% of positive scrapes in 2009–2013 and 2014–2018, respectively, without significant statistical differences in both periods. The same was observed with fungi (11% and 6.11% in 2009–2013 and 2014–2018, respectively) and virus (2% and 3.60% in 2009–2013 and 2014–2018, respectively). *Candida* specimens, *Fusarium* specimens, *and Aspergillus* specimens accounted for 4.18%, 1.46%, and 1.26% of all positive scrapes. The distribution of microbial profile is shown in Table 1.

We identified at least one ophthalmologic risk factor in 410 patients (85.77%). Additional systemic disorders were present in 17.57% (*n* = 84). Trauma and contact lens wear were the most common risk factors found, accounting for 34.94% (*n* = 167) and 33.47% (*n* = 160) of cases. The distribution of the risk factors is demonstrated in Table 2.

Sensitivity testing was not performed for *Corynebacterium macginleyi*, as the laboratory considered it as a contaminant, but it was performed in all the remaining bacterial isolates.

Sensitivity to fluoroquinolones and aminoglycosides was tested in all bacterial isolates, presenting values of 96.66% and 98.12%, respectively, with no significant statistical differences between the periods analyzed. The sensitivity to antibiotics is demonstrated in Table 3.

*Staphylococcus aureus* showed a high sensitivity profile to fluoroquinolones (ciprofloxacin, moxifloxacin, and levofloxacin) and to aminoglycosides (gentamicin and tobramycin), 89.41% and 98.82%, respectively. Two cases were methicillin-resistant *Staphylococcus aureus* (MRSA), being sensitive to vancomycin and linezolid only. All *Streptococcus pneumonia* isolates were sensitive to fluoroquinolones and aminoglycosides, and only a small proportion (4.44%) was resistant to tetracyclines. 85.72% of *Staphylococcus epidermidis* isolates were sensitive to fluoroquinolones and the same proportion to aminoglycosides, but resistance to tetracycline was observed in 38.09% of cases.

*Pseudomonas aeruginosa* presented a sensitivity of 95.35% to fluoroquinolones and aminoglycosides, and 27.91% demonstrated resistance to cotrimoxazole. Besides, 4.35% of cases showed multidrug resistance (resistance to at least three different classes of antibiotics), being sensitive exclusively to cefepime and colistin. All *Serratia* specimens and *Moraxella catarrhalis* isolates were sensitive to fluoroquinolones and aminoglycosides. No differences were observed in sensitivity to antibiotics in both periods analyzed.

## 4. Discussion/Conclusion

Considering the widespread use of broad-spectrum antibiotics, antibiotic resistance among bacterial microorganisms in general is a real concern nowadays, and ocular pathogens are no exception. Therefore, constant local updates of microbiological profiles and antibiotic resistance patterns are crucial to wisely choose the most appropriate topical treatment to ocular infections.

Infectious keratitis is a serious condition that can lead to a poor visual outcome or even loss of the eyeball. The diversity of the clinical presentation constitutes a huge challenge to an accurate diagnosis and treatment. Untreated keratitis can lead to opacification and/or perforation of the cornea, as well as to other equally fearsome complications such as secondary glaucoma, corneal thinning, uveitis, and endophthalmitis. Due to this rapidly progressive and potentially devastating course, an appropriate treatment with effective empirical topical antibiotics is mandatory.

It is well known that the epidemiological pattern of infectious keratitis significantly varies between countries and even among regions of the same country [8–13]. Therefore, determining the local microbiological profile, of one specific region or country, as well as its antibiotic resistance patterns and trends over the years is of major interest to achieve an effective therapeutic strategy [13–15].

In this study, we analyzed a total of 1360 corneal scrapes obtained from patients diagnosed with infectious keratitis, admitted to a tertiary hospital in Portugal.

We obtained a 35.1% (*n* = 478/1360) culture-positive rate, a result in agreement with similar studies published in the literature [14, 16, 17].

In our study, bacteria were the agent isolated in the vast majority of cases. Gram-positive bacteria were identified in 53.34% of the positive cultures, a value that is similar to other studies already published [14, 17]. However, in countries like the United Kingdom or New Zealand, proportions of 38.9% and 83%, respectively, have been reported [16, 18]. A Canadian study reported a decreasing trend in Gram-positive pathogens, probably due to the generalization of contact lens usage [13]. However, in our study, no statistically significant differences between Gram-positive and Gram-negative bacteria were observed between the 2 analyzed periods.

The most frequent agent identified was *Corynebacterium macginleyi* (18.41%), followed by *Staphylococcus aureus* (17.78%), *Streptococcus pneumoniae*, (9.41%) and *Pseudomonas aeruginosa* (9.00%). In the literature, some studies showed *Pseudomonas aeruginosa* as the most frequent agent [13, 14, 16, 18], while in other studies, *Staphylococcus aureus* is the main causal agent [13, 14, 16]. In our hospital, *Corynebacterium macginleyi* was considered by the Microbiological Department as a scrape contaminant. Nevertheless, it seems to actively contribute to the pathophysiology of blepharitis, and thus, it can potentially play a role in the genesis of infectious keratitis, particularly if some risk factors are present [19]. For this reason, its importance as a causative agent of infections has been progressively taken into account [20–22].

*Acanthamoeba* accounted for 12.13% of positive scrapes, and this value is located between the ranges published in the literature (1.6% and 16.9%) [1, 23].

Fungi represented 8.16% of positive scrapes, *Candida* spp. being the most frequent. However, the incidence of fungi is much higher in some countries such as Brazil (30%) or India (23–36%) since these microbiological agents are more frequent in tropical and subtropical regions than in the temperate regions [1, 24, 25].

Globally, fluoroquinolone sensitivity was high (96.66%), and the same was observed with aminoglycoside sensitivity (98.12%). Although these results are similar to several other series already published, [13–16, 18] resistances to fluoroquinolone and aminoglycosides of about 15% and 22% have been reported in Switzerland [26]. In our region, fluoroquinolones and aminoglycosides are almost exclusively prescribed by ophthalmologists. In primary health care, chloramphenicol (eye drops and ointment) and oxytetracycline (ointment) are the most widely used drugs. This probably explains why patients naive to infectious keratitis have low resistance rates.

Infectious keratitis caused by MRSA is an alarming issue all around the world due to its poor response to conventional antibiotic treatment [27–29]. Our rate of MRSA was 0.42%, lower than other reported series [2]. Like in other studies previously published, we observed that the rate of MRSA sensitivity to vancomycin was 100%, and therefore, vancomycin is a drug of inestimable value for the treatment of MRSA infections [30–34].

In our Microbiological Department, *Corynebacterium macginley* was considered a scrape contaminant. In the first reports in the literature, this agent was highly sensitive to common topical antibiotics, but this scenario has now changed, and high levels of resistance to fluoroquinolones have been already reported [35–37].

Some of the differences found in our study, compared to others already published, can be explained by factors such as the geographic location and associated climate and the extent and type of empirical antimicrobial therapy instituted before corneal scrapes. In our region, the most common bacteria are *Staphylococcus aureus, Streptococcus pneumoniae*, *and Pseudomonas aeruginosa*, and they showed high sensitivity rates to first-line antibiotics, without any modification or emergence of antibiotic resistance trends during the 10 years of the study. For this reason, we decided to maintain the same internal protocol in our tertiary centre.

## Figures and Tables

**Table 1:** Distribution of microbial profile.

	*n* = 478	%
Bacteria	367	76.78
Gram positive	255	53.34
*Corynebacterium macginleyi*	88	18.41
*Staphylococcus aureus*	85	17.78
*Streptococcus pneumoniae*	45	9.41
*Staphylococcus epidermis*	21	4.39
*Streptococcus mitis/oralis*	7	1.46
*Streptococcus viridans*	7	1.46
*Streptococcus pyogenes*	1	0.21
*Kocuria rosea*	1	0.21
Gram negative	112	23.44
*Pseudomonas aeruginosa*	43	9.00
*Serratia* specimens	27	5.65
*Moraxella* spp.	20	4.18
*Haemophilus influenzae*	7	1.46
*Enterobacter cloacae*	4	0.84
*Morganella morganii*	3	0.63
*Proteus mirabilis*	3	0.63
*Aeromonas hydrophila*	2	0.42
*Klebsiella* spp.	1	0.21
*Kingella kingae*	1	0.21
*Stenotrophomonas maltophilia*	1	0.21

*Acanthamoeba*	**58**	**12**.**13**

Fungi	**39**	**8**.**16**
*Candida* spp.	20	4.18
*Fusarium* spp.	7	1.46
*Aspergillus* spp.	6	1.26
*Paecilomyces* spp.	3	0.63
*Scedosporium* spp.	2	0.42
*Mucor*	1	0.21

Virus	**14**	**2**.**93**

**Table 2:** Risk factors.

	*n*	%
Local	**410**	**85**.**77**
Ocular trauma	167	34.94
Contact lens use	160	33.47
Blepharitis	20	4.18
Ocular infection	19	3.97
Exposure keratopathy	13	2.72
Dry eye	10	2.09
Bullous/band keratopathy	8	1.67
Recurrent erosion	7	1.46
Trichiasis	3	0.63
Herpetic keratitis history	3	0.63

Systemic	**105**	**21**.**97**
Diabetes mellitus	60	12.55
Poor systemic status/multiple comorbidities	14	2.93
Mucocutaneal disease	13	2.72
Autoimmune disease under immunosuppressant	6	1.26
Neoplasia	6	1.26
HIV	3	0.63
Drugs	3	0.63

**Table 3:** Sensitivity to fluoroquinolones and aminoglycosides.

Bacteria		*n*	Sensitivity to fluoroquinolones (%)	Sensitivity to aminoglycosides (%)
Gram positive	*Staphylococcus aureus*	85	89.41	98.82
	*Streptococcus pneumoniae*	45	100	100
	*Staphylococcus epidermis*	21	85.72	85.72
	*Streptococcus mitis/oralis*	7	100	71.43
	*Streptococcus viridans*	7	100	71.43

Gram negative	*Pseudomonas aeruginosa*	43	95.35	95.35
	*Serratia* specimens	27	100	100
	*Moraxella* spp.	20	100	100
	*Haemophilus influenzae*	7	100	100

## Data Availability

The data used to support the findings of this study are available from the corresponding author upon request.
